# Internet-based media coverage on dengue in Sri Lanka between 2007 and 2015

**DOI:** 10.3402/gha.v9.31620

**Published:** 2016-05-12

**Authors:** Annelies Wilder-Smith, Emily Cohn, David C. Lloyd, Yesim Tozan, John S. Brownstein

**Affiliations:** 1 Lee Kong Chian School of Medicine, Nanyang Technological University, Singapore; 2 Department of Public Health and Clinical Medicine, Epidemiology and Global Health, Umeå University, Umeå, Sweden; 3 Institute of Public Health, University of Heidelberg, Heidelberg, Germany; 4 Boston Children's Hospital, Boston, MA, USA; 5 College of Global Public Health, New York University, New York, NY, USA

**Keywords:** dengue, media, news coverage, Internet, emergence of dengue, awareness about dengue, malaria, influenza, Healthmap, Sri Lanka

## Abstract

**Background:**

Internet-based media coverage to explore the extent of awareness of a disease and perceived severity of an outbreak at a national level can be used for early outbreak detection. Dengue has emerged as a major public health problem in Sri Lanka since 2009.

**Objective:**

To compare Internet references to dengue in Sri Lana with references to other diseases (malaria and influenza) in Sri Lanka and to compare Internet references to dengue in Sri Lanka with notified cases of dengue in Sri Lanka.

**Design:**

We examined Internet-based news media articles on dengue queried from HealthMap for Sri Lanka, for the period January 2007 to November 2015. For comparative purposes, we compared hits on dengue with hits on influenza and malaria.

**Results:**

There were 565 hits on dengue between 2007 and 2015, with a rapid rise in 2009 and followed by a rising trend ever since. These hits were highly correlated with the national epidemiological trend of dengue. The volume of digital media coverage of dengue was much higher than of influenza and malaria.

**Conclusions:**

Dengue in Sri Lanka is receiving increasing media attention. Our findings underpin previous claims that digital media reports reflect national epidemiological trends, both in annual trends and inter-annual seasonal variation, thus acting as proxy biosurveillance to provide early warning and situation awareness of emerging infectious diseases.

## Introduction

Dengue is a mosquito-borne viral disease that has emerged as a major public health problem in many countries of the tropics and subtropics (1). The estimated increase in age-standardized incidence and prevalence rates was about 447% during the period 1990 to 2013 according to the recent global burden of disease study, by far the largest increase among all other common infectious diseases, including HIV and malaria, in this time period (2). Sri Lanka, an island nation in South Asia with a population size of 20,359,439 (2012 census), has seen dengue outbreaks of increasing frequency and magnitude since the early 2000s (3, 4), and dengue now poses a substantial economic and disease burden to the population and healthcare system (5–7). Laboratory-enhanced surveillance in Colombo, for example, showed that around 43% of all hospitalized undifferentiated fevers is due to dengue (7). The highest disease incidence is found in highly populated and urbanized areas of the island, especially in the capital city of Colombo.

In terms of outbreak detection and response, the International Health Regulations have shifted their emphasis from public health surveillance to situational awareness, particularly in developing countries (8). The extent of news coverage often reflects the advent of new outbreaks or the worsening of epidemics. As global media companies make news increasingly available online, it is possible to use digitalized media coverage to explore the extent of public awareness of a disease and even use such coverage as a proxy for outbreak detection and perceived severity of an outbreak at a national level. With rapidly increasing global Internet use, novel Internet-based disease monitoring tools have emerged recently. Google Trends was a novel free tool that allowed users to interact with Internet search data and was increasingly used to track real-time infectious disease activity (9, 10). For example, Google Flu Trends, a currently discontinued web-based surveillance tool, could detect regional outbreaks of influenza 7–10 days before conventional Centers for Disease Control and Prevention surveillance (11). Google Dengue Trends, a similar surveillance tool that was developed later, used real-time Google search query data to create an index of dengue incidence; initial studies showed Google search queries to be a close proxy for traditional dengue surveillance in many countries (12).

In this study, we performed search queries using HealthMap (www.healthmap.org/) – a global infectious disease outbreak monitoring and surveillance tool, which utilizes online informal sources, such as online news aggregators, eyewitness reports, expert-curated discussions, and validated official reports, to identify trends in digital media reporting on dengue in Sri Lanka between 2007 and 2015. For comparative purposes, we also looked at reporting on influenza, including H1N1, and malaria over the same time period.

HealthMap has already been used to examine trends and patterns in health in different contexts (13–15).

## Design

The HealthMap global infectious disease news monitoring system draws on a number of online data sources, including ProMED-mail (the Program for Monitoring Emerging Diseases, a program of the International Society for Infectious Diseases), the World Health Organization, the World Organisation for Animal health, the Food and Agriculture Organization of the United Nations, and Eurosurveillance from the European Centre for Disease Prevention and Control. It also aggregates information from VeriSign, Wildlife Data Integration Network from the University of Wisconsin, Baidu News, SOSO Info, and Google News Archives. Google News Archives is a commercial news aggregator that provides access to a broad range of global English language digital media reporting and has the capacity to run news searches according to specific keywords and dates (12).

All digital media news articles where the location had been assigned to ‘Sri Lanka’ and pertained to the disease ‘Dengue’, ‘Malaria’, ‘Influenza’, or ‘Influenza H1N1’ were extracted from the HealthMap database for the time period between January 2007 and November 2015. News articles were searched in the following languages: English, French, Spanish, German, Russian, Italian, Portuguese, Arabic, Chinese, Vietnamese, Korean, Thai, Japanese, and Bahasa (Indonesian/Malaysian). HealthMap does not have functionality in Sinhala or Tamil, the two official languages of Sri Lanka.

The number of notified dengue cases over the study period was obtained from the National Communicable Disease Surveillance System database run by the central Epidemiology Unit attached to the Ministry of Health (www.epid.gov.lk).

## Results

There were 565 news article hits drawn from the HealthMap system between 2007 and 2015. Table 1 shows the languages in which the new articles appeared for the four diseases in reference to Sri Lanka. These counts do not include duplicate articles, where multiple websites picked up the original article and reprinted it.

**Table 1:** Total number of Internet reports on each disease in relation to Sri Lanka by language during the period 2007–2015

Language	Dengue	Malaria	Influenza	H1N1 influenza
English	338	37	28	97
Chinese	31	0	2	1
French	2	0	1	4
Portuguese	10	0	0	2
Spanish	3	1	0	0
Russian	5	0	0	0
Vietnamese	0	0	0	1
Indonesian	1	0	0	0
Japanese	1	0	0	0
Total	391	38	31	105

**Fig. 1:**
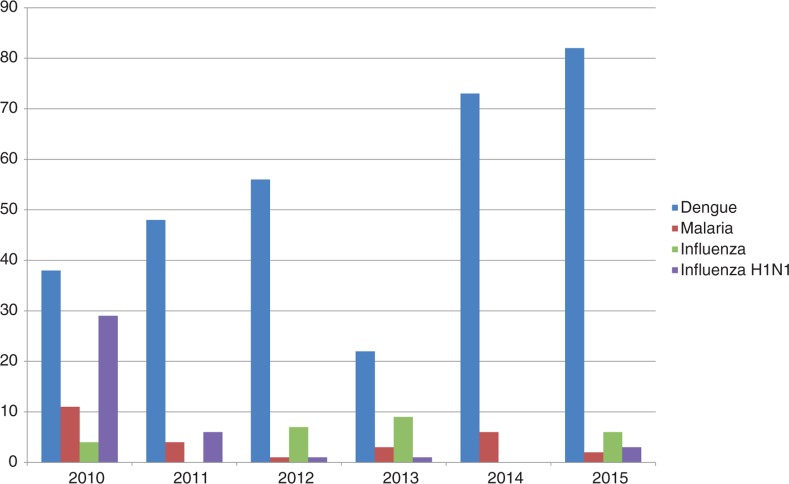
Number of Internet reports on Sri Lanka by year and disease, 2007–2015.

**Fig. 2:**
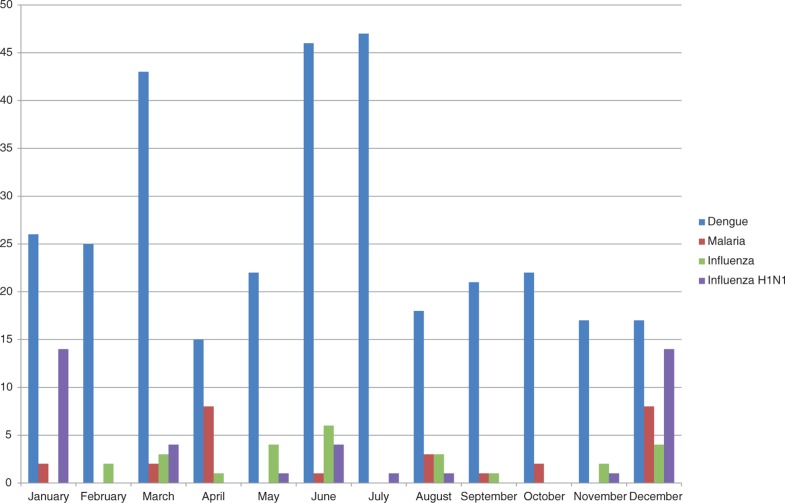
Number of Internet reports for each disease in relation to Sri Lanka by month, during the period January 2007 to November 2015.

**Fig. 3:**
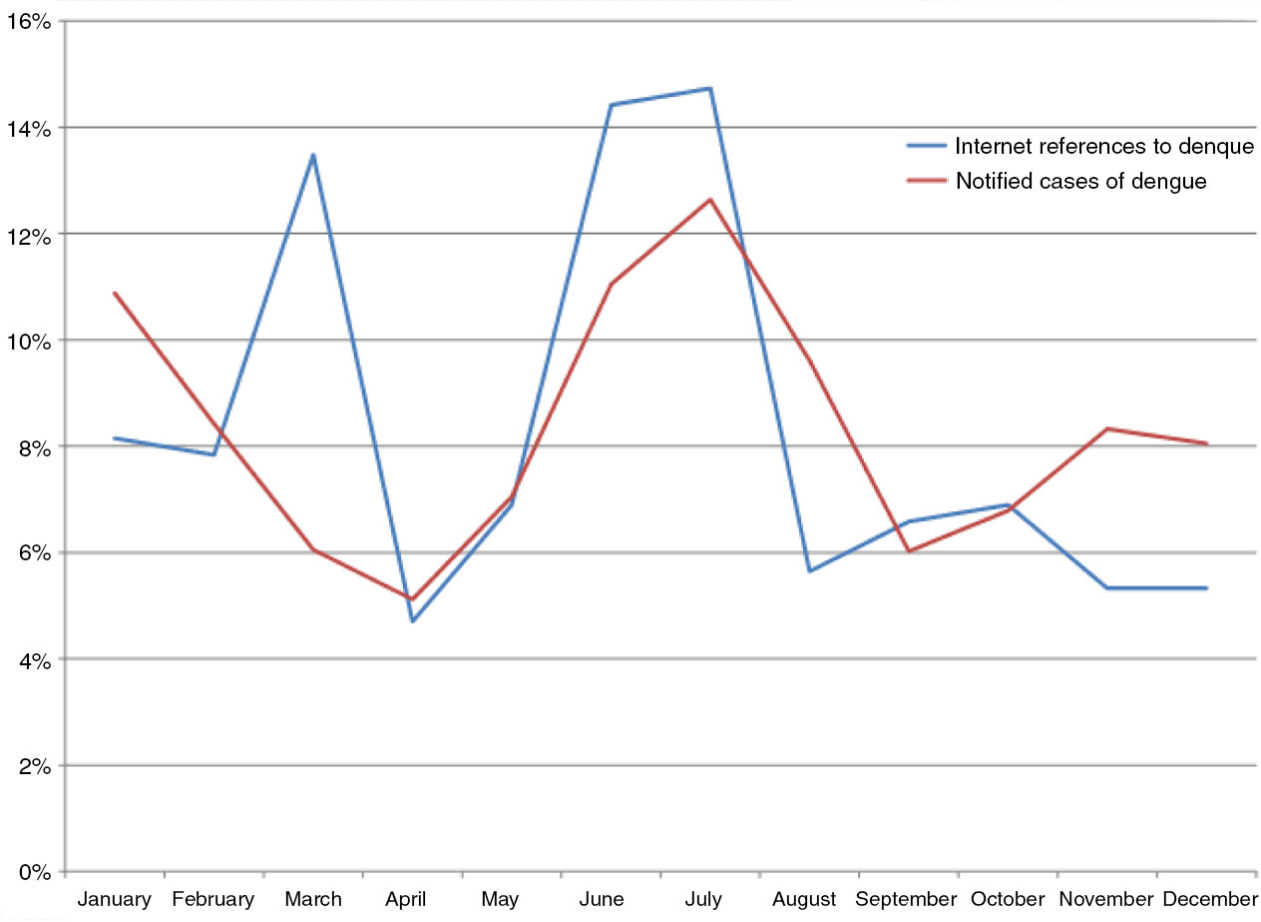
Monthly comparison of Internet references on dengue with notified cases of dengue in Sri Lanka, summed across for the period January 2010 to November 2015.

Figure 1 shows the number of news articles each year that mentioned Sri Lanka and the four diseases. Clearly, there was considerable concern about the H1N1 strain of influenza in 2009, as reflected by a peak in media reports on H1N1 for that year, but not the following years. Coinciding with the dengue outbreak that started in 2009, the news articles on dengue skyrocketed in 2009 and have remained high ever since. Figure 2 shows the trends averaged by month to present dengue seasonality within a year. As the Epidemiology Unit of the Ministry of Health Sri Lanka did not publish notified dengue cases before 2010, we could only use the data from January 2010 to November 2015 to correlate media reports of dengue with notified dengue cases. Figure 3 compares the percentage of notified cases in each month summed over the period January 2010 to November 2015 with the percentage of news-media references to dengue in Sri Lanka by month. Pearson correlation between Internet references and dengue cases is 0.52 with 11 degrees of freedom, *p*<0.1.

## Conclusions

Our findings highlight the substantial increase in digital media reports on dengue between 2007 and 2015. This can be partially explained by the increasing availability of digital media, but the increase mainly appears to be a reflection of the rapidly increasing incidence of dengue in Sri Lanka. Although the four dengue virus (DENV) serotypes have been co-circulating in Sri Lanka for more than 30 years, the emergence of new clades of DENV-3 in 1989 and 2000 coincided with major increases in the number of notified dengue cases (5). In 2009–2013, Sri Lanka experienced an even larger exponential increase in dengue cases, with an average of 35,000 cases per year and an incidence of 175 per 100,000 population reported nationally (www.denguevaccines.org/dengue-sri-lanka-burden-challenges-and-prevention-strategies-exclusive-interview-dr-hasitha-tissera). The abrupt increase in dengue cases in 2009 is paralleled by the abrupt rise in digital media reports on dengue. The intense media coverage on dengue also reflects the government's focus on this disease. In 2010, in response to the rapid rise in dengue cases seen since 2009, Sri Lanka made dengue control a national priority (www.southasia.oneworld.net/news/sri-lanka-makes-dengue-control-a-national-priority#.VppFZ1JrbNo). Public health authorities launched massive community-level prevention campaigns targeting dengue using billboards and television advertisements. The Ministry of Health initiated national dengue prevention weeks and launched efforts to clean up breeding grounds, assisted by armed and police forces.

Media interest for dengue in Sri Lanka far exceeds that for malaria. This is because the malaria eradication campaign achieved a dramatic reduction in malaria cases from 210,039 in 2000 to 23 cases in 2012–the lowest number of malaria cases reported since 1963 (www.malariacampaign.gov.lk/precentation/Home.aspx#sthash.AqMFLfI7.dpuf). The increase in dengue cases concomitant with a rapid decrease in malaria cases is also seen in many other Asian countries that have experienced rapid urbanization. Population growth, increased population density, and unplanned urbanization seem to be factors that drive the proliferation of vector breeding sites for the Aedes mosquito and hence dengue (16, 17), in contrast to Anopheles mosquitoes for malaria.

The media interest for influenza was relatively low, similar to that for malaria. Only in 2009, H1N1 influenza in Sri Lanka received massive media coverage, plausibly as a response to the World Health Organization declaring H1N1 an international public health emergency. Despite the high public interest in H1N1 influenza in 2009, as reflected by a peak in media reporting in that year, reports on influenza rapidly declined thereafter, while reporting on dengue was similarly high and remained high. Overall, digital media coverage of influenza (other than H1N1) in Sri Lanka is substantially lower than that of dengue.

Our study used data from HealthMap, which does not include Google searches, but rather relies on digital media sources and official body reports, supplemented by Twitter feeds. Although our primary objective was to describe digital media interest on dengue in Sri Lanka, our data also allowed us to examine the extent to which media interest reflects true dengue incidence. The trend line of media reports reflects the national epidemiology, with a sharp increase in the year 2009 and sustained outbreaks ever since. Also the seasonal variation (per month) of media reports on dengue parallels that of the seasonal variation of dengue with two annual peaks. Since the mosquito vectors that carry dengue also transmit Zika virus and chikungunya, this media coverage could be relevant to these diseases.

HealthMap does not include digital media reports in Sinhala or Tamil, the two official languages of Sri Lanka. This is clearly a limitation of our study. However, English is a commonly spoken language throughout Sri Lanka, and Sri Lanka has an active English language media. Therefore, it is not surprising that we found many media reports in English language. The website *onlinenewspapers.com* lists 81 online newspapers in Sri Lanka, of which 47 are in English or have English language versions.

In conclusion, dengue in Sri Lanka has received substantially higher attention in digital media than influenza and malaria over the past decade. This media attention was accentuated from 2009 onwards and remained high for several years. Our findings also underpin previous claims that digital media reporting reflects national epidemiological trends in both annual trends and inter-annual seasonal variation, thus acting as a proxy biosurveillance indicator to provide early warning and situation awareness of emerging diseases or new or escalating disease outbreaks (18).

## Paper context

Internet-based media coverage to explore the extent of awareness of a disease and perceived severity of an outbreak at a national level can be used for early outbreak detection. We examined Internet-based news media articles on dengue queried from HealthMap for Sri Lanka from 2007 to 2015, and found that digital media reports reflected national epidemiological trends, both annually and interannually. Thus, digital media coverage can act as proxy biosurveilance for dengue.
